# Mechanisms of Metastatic Tumor Dormancy

**DOI:** 10.3390/jcm2030136

**Published:** 2013-09-23

**Authors:** Mary Osisami, Evan T. Keller

**Affiliations:** Department of Urology, University of Michigan Medical School, 5111 CCGC1500 E. Medical Center, Ann Arbor, MI 48109-0940, USA; E-Mail: mosisami@umich.edu

**Keywords:** dormancy, tumor mass dormancy, tumor cell dormancy

## Abstract

Tumor metastasis can occur years after an apparent cure due to a phenomenon known as metastatic tumor dormancy; in which tumor masses or individual tumor cells are growth restricted for extended periods of time. This period of dormancy is induced and maintained by several mechanisms, including: (1) Tumor microenvironment factors such as cytokine expression, immunosurveillance and angiogenesis; (2) Metastasis suppressor gene activity; and (3) Cancer therapeutics. Disseminated tumor cells (DTC) are the key cells that result in dormant tumors. However, many challenges exist towards isolating DTCs for mechanistic studies. The main DTC that may represent the dormant cell is the cancer stem cells (CSC) as they have a slow proliferation rate. In addition to limited knowledge regarding induction of tumor dormancy, there are large gaps in knowledge regarding how tumors escape from dormancy. Emerging research into cancer stem cells, immunotherapy, and metastasis suppressor genes, may lead to new approaches for targeted anti-metastatic therapy to prevent dormancy escape. Overall, an enhanced understanding of tumor dormancy is critical for better targeting and treatment of patients to prevent cancer recurrence.

## 1. Introduction

The majority of cancer related deaths are due to metastatic outgrowths of the primary tumor mass that develop years to decades after apparent cures. Metastatic spread of tumors is a well-coordinated sequence of events, where cells shed from primary tumors, enter blood circulation, and spread to distant organs [1,2]. This process is, however, highly inefficient, where the majority of cells are predicted to die upon dissemination. Some disseminated tumor cells (DTC) will immediately begin to proliferate and colonize the new environment, but some DTC, while still viable, will enter a growth arrested state [3,4,5]. These growth arrested cells can remain viable and clinically undetectable for extended periods of time and are termed dormant cells. The dormant cells can awaken years later and resume proliferation and colonization even after the presumably successful treatment of the primary tumor [3,4,5]. In addition to a proliferation-arrested state (G0/G1 arrest) clinical dormancy may be due to micro-metastases where active proliferation is counterbalanced by apoptosis [6]. These metastatic growths are usually more malignant than the primary tumor, having acquired the ability to circumvent conventional therapies and growth barriers from non-permissive microenvironments. 

## 2. Tumor Cell Dormancy

Tumor cell dormancy is characterized by solitary cells existing in a quiescent like state accompanied by decreased expression of proliferation markers [6]. Tumor cell dormancy is caused by several events including, microenvironment induced stress, transcriptional program from the primary tumor, and even drug therapies for primary tumor treatment. There has been debate as whether or not this state is quiescence or a reversible senescence [6,7]. Quiescence and senescence are mechanisms to induce cell cycle arrest and therefore could lead to tumor dormancy. Quiescence is defined as reversible cell cycle arrest, while senescence is permanent cell cycle arrest [8]; since senescence is permanent proliferation arrest, it is assumed that tumor cells have evolved the ability to bypass senescence mechanism. Perhaps a combination of both pathways leads to tumor cell dormancy [7].

## 3. Microenvironment Induced Dormancy

The microenvironment can have a very profound effect on the ability of tumor cells to develop into clinically relevant tumors. This was first hypothesized by Stephen Paget who, in his seed and soil theory, theorized that metastatic tumors (seed) will only grow in microenvironments (soil) for which they are suited [9]. The microenvironment is in direct contact with the tumors cells and thus acts as a critical source of vital signals needed for tumor cell survival and proliferation [10]; adapting to the microenvironment is an essential step in successful metastatic tumor growth. While some tumors have a predisposition towards metastasizing to specific organs [2,11,12,13,14], they may not be able to immediately colonize the new region due to inefficient interactions with the microenvironment [6]. DTCs may encounter a new environment in which they are not compatible with and therefore cannot fully engage the extracellular matrix. For example, Barkan *et al.* showed that cells incapable of making cytoskeletal rearrangements to fully engage the microenvironment will enter into and remain in a dormant state until they can make the needed modifications [15]. Using breast cancer cell lines D2Al and D2.0R, which exhibit similar proliferation rates *in vitro*, have different characteristics *in vivo* D2.0R remain as single quiescent cells for extended periods of time, compared to D2A1 cells which remain dormant for a relatively short time and switch to form rapid growing masses, Barkan *et al.* showed that these cells differentiate in their ability to express fibronectin and therefore induce β-1 integrin signaling and cytoskeletal rearrangements [15]. Under these conditions, the microenvironment is interpreted as hostile, as the cells only have transient adhesion to the microenvironment, leading to the activation of stress response signaling such as, urokinase*-*type plasminogen activator receptor (uPAR) deactivation [15,16,17]. uPAR is a metastasis-associated receptor that leads to tumor growth through α5β1 integrin interactions [16]. Low uPAR signaling prevent DTCs from interacting with and activating B1 integrin and downstream signaling events, including cytoskeletal dynamics, reducing microenvironmental interactions [6,15,18]. In addition to down regulation of uPAR signaling, microenvironment-induced stress also leads to p38 activation and ERK1/2 deactivation [19,20,21]. p38 activation has been shown to inhibit tumor progression as it implicated in promoting growth arrest, by activating p53 andp16 signaling, and down regulating cyclin D1 [22,23,24,25]. It has also been implicated in reducing the expression and activation of mitogenic signaling of ERK1/2 [19]. The ratio of ERK1/2 and p38, activation has been shown to predict if a tumor cell will proliferate or enter a dormant state upon dissemination, with a high ratio suggesting proliferation and a low ratio suggesting dormancy [20].

Microenvironment induced stress may induce the expression or activation of metastasis suppressor genes (MSGs) [26]. MSGs are genes that prevent the formation of metastases, while having little to no effect on primary tumor formation. MSGs act on a wide range of cellular processes to inhibit metastatic growth including activation of signaling pathways which promote dormancy through cell cycle arrest or deactivating signaling pathways which promote cell proliferation (reviewed in [27]). The MSGs mitogen-activated proteins kinase-kinase (MKK) 4 and MKK6 have been shown to activate p38 signaling [28,29]; with MKK4 also activating and the cyclin-dependent kinase inhibitor p21, inducing cell cycle arrest. MSG N-myc downstream-regulated gene 1 (NDRG1), has been implicated in regulating oncogenic signaling pathways of TGF-β, PI3K, and Ras [30].

In addition to activating stress responses through inefficient adhesion/interaction, there are reports that suggest that microenvironments, as part of their normal activity, can secrete factors that are anti-proliferative to DTCs. For example bone marrow stromal cells secrete bone morphogenic protein 7 (BMP7), which has been shown to induce dormancy in prostate cancer tumor cells [31]. The secretion of BMP7 leads to the increase of the metastasis suppressor gene NDGR1, which subsequently leads to an increase in p38 activation, cell cycle inhibitor p21 expression and ultimately cell cycle arrest [31]. Another example, also within the bone, occurs with the secretion of growth arrest-specific 6 (GAS6) by osteoblasts and tumor cells, which induces prostate cancer tumor cell dormancy [32]. Shiozawa *et al.* showed that GAS6 expression within the bone leads to a decrease in prostate cancer cell proliferation and an increase in chemoresistance [32]. Lim *et al.* showed that breast cancer cells in contact with bone stromal cells enter G0/G1 arrest by receiving proliferation-inhibiting microRNAs from the stromal cells, a phenomenon that is inhibited when gap junction intercellular communication is inhibited [33].

In addition to stressed induced MSG expression, some cells disseminate from the primary tumor with a gene expression profile that is prone to tumor dormancy. Recent studies have found gene expression signatures within primary tumors (in addition to ERK1/2 and p38 ratio) that predict if tumors will produce dormant cells with early or late reoccurrence [34,35]. Kim *et al.* using gene signatures identified in dormancy models of tumor cell quiescence and angiogenic failure, generated a 49-gene expression profile [34]. Using this gene profile, they have developed a scoring system to determine if tumor will produce late or early reoccurring tumors.

## 4. Treatment-Induced Dormancy

Tumor dormancy may arise as a response to cancer treatments [36,37,38,39]. The majority of treatments for cancer targets rapidly dividing cells. To circumvent drug induced death, some cancer cells will undergo cell cycle arrest/dormancy mechanisms that inhibit proliferation to survive. For example, ovarian tumor cells treated with farnesyl tranferase inhibitors (FTIs) undergo tumor dormancy by inducing autophagy [37]. Autophagy, the process of cellular organelle degradation to decrease cellular energy consumption and avoid apoptosis, occurs when cells experience prolonged periods of stress such as low nutrition, toxicity or to avoid anoikis [40,41,42]. This suggests that in order to survive a hostile environment and even drug treatment, tumor cells will induce autophagy, which has been reported to be the gateway to cell cycle arrest and tumor dormancy [42,43,44]. Some chemotherapeutic drugs, have been linked to an increase in p53 expression to induce senescence along with apoptosis in tumor cells [45]; however, there are reports that suggests that p53 induction can also lead to the induction of quiescence [46,47]. Tamoxifen exposure has also been shown to activate p38 [48]; which as mentioned above may lead to dormant cells. This suggests that chemotherapy may cause a subset of tumor cells to enter into quiescence and thus dormancy. Treatment induced dormancy may also be linked to cancer stem cells (CSCs), since these cells are slow cycling compared to the bulk of actively dividing cell within the tumor mass.

## 5. Cancer Stem Cells

CSCs represent a small population of cells within a tumor that are responsible for tumor maintenance, as they are fully capable of reconstituting a tumor, unlike the non-stem cell population within a tumor mass [49]. Like adult progenitor cells, these cells are predominately quiescent and may contribute to tumor dormancy, since they are largely resistant to majority of chemotherapies, which typically target rapidly dividing cells [37,50,51]. They can also become quiescent through co-opting target organ progenitor cell mechanism for quiescence, as demonstrated by Shiozawa *et al.* showed that prostate cancer cells are able to compete with hematopoietic stem cells [52]. After treatment, these cells are then free to slowly divide and rebuild the tumor leading to metastatic growth. As mentioned above, tumor dormancy can be a survival mechanism during therapy, with treatments able to specifically induce dormancy in CSCs [37]. Tumor cells may, as a survival mechanism to conventional drug treatments, spontaneously convert to CSCs. For example, it has been reported that non-stem tumor cells (NSTCs) may spontaneously convert to CSCs [53]. Specifically, Chaffer *et al*, showed mammary NSTCs (CD44^10^, CD24^+^) can spontaneously convert into mammary CSCs (CD44^hi^, CD24^−^), and give rise to both stem and non-stem cells [53]. This conflicts with the tenets of the CSC theory, that only CSCs can give rise to both CSCs and NSTCs [54,55]. However, there is some evidence that suggests that stemeness in tumorl cells may be transient, with any cell within a tumor population exhibiting stem-like qualities at any given moment [56]; with this conversion to the CSC phenotype is linked to epithelial-mesenchymal transition (EMT) [57]. Mani *et al.* demonstrated that forced induction of EMT leads to an increase in the expression of CSC markers and CSC properties [57]. As there are several lines of evidence that chemotherapy can lead to the induction of EMT [58,59,60], it is plausible treatment is enriching for or pushing cells into exhibiting stem-like properties, that would be capable of reconstituting a tumor at a later time point. 

## 6. Tumor Mass Dormancy

Unlike tumor cell dormancy, tumor mass dormancy arises from DTCs that are able to proliferate at the metastatic site, but do not continue to progress to a clinically apparent metastasis as their growth is limited due to insufficient angiogenesis or active immunosurveillance. These cells exist as micro-clusters of cells actively proliferating, but not able to grow beyond a few mm as the rate of proliferation is equal to the rate of apoptosis. These micro-masses, like solitary cells, may remain indolent for extended periods of time.

## 7. Immune System and Tumor Dormancy

It has been long known that the immune system can have profound effects on tumor formation and progression. An active role for the immune system in preventing tumorigenesis is seen in transplant recipients, who after immunosuppressive therapy, spontaneously develop tumors at a higher rate than the general population or develop tumors of donor origin; where donors have no history of cancer [61,62,63,64]. This was also demonstrated in experiments that showed tumor formation and progression was higher in immunodeficient mice *vs.* immunocompetent mice [65,66,67,68,69,70,71]. Performed mainly by cells of the adaptive immunity, the immune system contributes to dormancy of DTCs by eliminating highly immunogenic tumor cells through cytolysis (reviewed in [66,72,73,74]). Early in the immunoediting process, immune cells are highly intolerant of tumor cells effectively managing to suppress tumor cell growth [74]. However, as the process continues tumor cells with low immunogenicity or tumor specific antigen (TSA) expression begins to emerge, creating a “stale mate” between tumor cells and immune cells. As tumors cells proliferate, immune cells are killing high TSA expressing tumor cells at the same rate [73]. This was shown by transplanting “unedited” tumors from immunodeficient mice and placing them in immunocompetent mice [66]. In these experiments, the unedited tumors were quickly cleared in immunocompetent mice, suggesting that tumors developed in immunocompetent mice are less immunogenic than tumors developed in immunocompromised mice. This was also demonstrated by Kobel *et al.* where immunocompetent mice, treated with the carcinogen 3′-methylcholanthrene (MCA) at low doses, maintained occult tumors cells without tumor outgrowth for extended periods of time [75]. The mice developed overt tumors after treatment with monoclonal antibodies directed against components of the immune system in the same location as the MCA injections, which provides strong evidence of the ability of the immune system to maintain micro-masses in a clinically apparent dormant state. This was further demonstrated in the DA1-3b mouse leukemia model. In mice vaccinated with DA1-3b cells expressing CD40L or IL-12 followed by a live DA1-3b cell challenge, without overt, showed few dormant tumor cells; which were able to induce AML when isolated and injected into naive mice. This further supports the hypothesis that tumor dormancy can result from a population of tumor cells that persists in balance with the immune system [76].

Immune cells are also capable of inducing dormancy and preventing the aggressive outgrowth of metastatic tumors through non-cytotoxic methods [75,77,78,79]. Eyles *et al.* showed that CD8^+^ T-cells can prevent metastatic outgrowth in non-orthotopic organs, through cytostatic effects on disseminated tumor cells [74,80]. T-cells have been shown to inhibit cell cycle progression of tumor cells through IFN-γ and TNF mediated signaling, independent of cytotoxicity induction [75,77,78,79]. Muller-Herm *et al.* showed that IFN-γ producing TNFR^+^ CD4^+^ T-cells can inhibit tumor cell proliferation and angiogenesis [77]. The immune system also quickly clears pathogenic infections, which limits inflammatory responses prevent that can ultimately induce tumor cell growth and even induce angiogenesis (reviewed in [74]). 

There is some controversy surrounding the role of the immune system in metastatic tumor dormancy, since, in theory, metastatic tumors cells should have acquired the needed mutations to circumvent the immune targeting [7]. However, there is evidence to suggest that primary tumors induce changes in the microenvironment, which leads to non-global immune cell tolerance in the primary site that is not granted to metastatic cells in a new microenvironment [81,82]. This was demonstrated in several experiments were mice injected with tumors cells eventually have actively growing tumors, but reject secondary challenges with the same tumor cells at different sites [83,84,85]. In addition to tumor cell killing and inhibition of tumor cell proliferation, immune cells, may also play a role in preventing angiogenesis, as natural killer cells can secrete anti-angiogenic factors [86].

## 8. Angiogenesis and Tumor Dormancy

As tumor cells proliferate and develop into clinically apparent masses, they must recruit and sustain their own blood supply through a process called angiogenesis [87]. Defined as one of the “hallmarks of cancer”, angiogenesis refers to the sprouting of, or recruitment of new blood vessels from existing vasculature in response to lack of oxygen and nutrients [87]. After reaching approximately 1–2 mm, the tumor becomes deficient in oxygen and nutrients as the nutrients in the microenvironment can no longer support the needs of the micro-metastases [88]. While proliferation competent, tumor cells may not be able to induce angiogenesis due to failure to express or induce the expression of factors necessary for angiogenesis to occur. Angiogenesis is controlled through pro- and anti-angiogenic factors such as vascular endothelial growth factor (VEGF) and angiostatin, respectively, within the tumor microenvironment. It is plausible that due to early shedding of tumor cells from the primary tumor, DTCs may not have acquired the ability to induce angiogenesis in its new microenvironment. In addition to expression of pro-angiogenic factors, angiogenesis is dependent on the proliferation and recruitment of endothelial cells to nearby vasculature in the tumor microenvironment. Png *et al.* showed that tumor cells expressing microRNA cluster 126 (miR-126) inhibit the recruitment of endothelial cells to the tumor site, through blocking GAS6/MER signaling [89]. They also showed that these cells were proliferation competent and angiogenic competent, when co-transplanted with endothelial cells [89]. Straune *et al.* also showed that tumor cells with low heat shock protein 27 (HSP27) expression remain non-angiogenic and dormant for extended periods of time in part due to inhibition of endothelial cell proliferation [90]. HSP27 expression lead to an increase in microvessels and proliferating cells within the microvessels [90].

## 9. Escape from Tumor Dormancy

As mentioned above the microenvironment can have profound effects on the fate of DTCs, stimulating dormancy induction as well as escape from dormancy. Changes in the microenvironment, especially age-related changes, can induce tumor cells to escape from dormancy. As a host ages, more of their cells enter senescence [91,92,93,94,95], which can lead to an increase the activation of dormant cells. Senescent cells, while proliferation-inhibited, may become highly secretory; secreting high levels of cytokines, chemokines, growth factors and proteases in a phenomenon known as senescence-associated secretory phenotype (SASP) [96]. The secretome includes strong pro-proliferation molecules and pro-inflammatory molecules as well as pro-angiogenic molecules creating an environment that stimulates tumor cell proliferation, and promotes angiogenesis (reviewed in [96]). Changes within the microenvironment can also lead to the mobilization and activation of CSCs. As mentioned earlier, CSCs can co-opt target organ progenitor cell mechanisms for quiescence. As a host ages there are also changes to tissue stem cell niches, leading to increased growth and mobilization of stem cells [97]. This can also lead to the activation and mobilization of CSCs and subsequently, tumor reoccurrence.

In addition to age-related changes within a host, diet/and medications can tumor growth and dormancy. A poor diet can lead to an increase in adipose tissue and obesity; which has been linked to tumor reoccurrence in breast cancer [98]. Adipocytes have been shown to cause inflammation responses through secretion of MMP11, as well as pro-inflammatory cytokines IL-6 and IL-1β [99]. Adipose tissue is linked to estrogen secretion; which can also lead to the stimulation of tumor cell proliferation [100].

Medications, as mentioned above, may have profound effects on dormancy in tumor cells. Drugs that act as soluble epoxide hydrolase inhibitors to increase vascular epoxyeicosatrienoic acids (EETs), are in clinical trials for use in treatment of cardiovascular disease, and may induce tumor dormancy escape through stimulating angiogenesis [101,102]; allowing tumor cells to proliferate beyond micro-metastases.

As mentioned earlier, immune cells target tumor cells with high levels of TSA expression [66,72,73,74]. As the immune system eliminates cells with high levels of tumor specific antigen expression, they are selecting for cells with low to no expression of TSA; giving rise to a population of cells that have the ability to grow without interference from immune cells [103]. In addition to tumor evolution through selection for less immunogenic cells, tumor cells may interact with the microenvironment to create an environment that inhibits the recruitment of immune cells, giving tumor cells selective permission for growth in a particular environment [72,83,84,85]. Cells may also attain the necessary mutations, while dormant, colonize the new microenvironment or induce an angiogenic switch [104,105].

## 10. Conclusions and Future Directions

**Figure 1:**
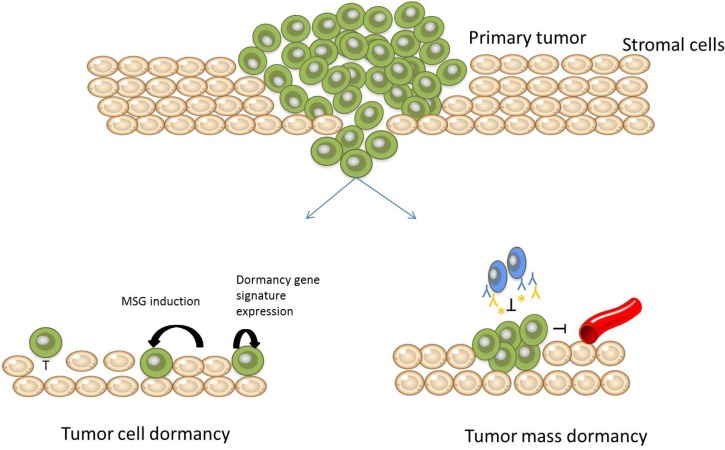
Schematic of metastatic tumor dormancy. Some tumor cells will leave the primary tumor without the ability to proliferate in the new microenvironment and remain as solitary cells (tumor cell dormancy), due to microenvironmental-induced stress, microenvironment incompatibility or even a gene expression profile that is prone to dormancy. Some tumor cells can leave the primary site with the ability to proliferate in the new site, but cannot grow beyond a few mm (tumor mass dormancy) due to immunosurveillance or angiogenic failure.

There are multiple factors which contribute to tumor dormancy (Figure 1). The microenvironment provides signals to tumor cells which can confer growth arrest through the induction of stress signaling such as p38 activation. The microenvironment can also inhibit the activation of angiogenesis, a necessary step for sustained metastatic growth. The immune system can also lead to dormancy by inhibiting the net proliferation of tumor cells, but not necessarily tumor cell shedding. After an apparent cure, some patients will have circulating tumor cells (CTCs) within their blood system without disease presentation, suggesting that they have dormant tumors; however these cells or dormant tumors may or may not become clinically apparent within their lifetime.

Despite a myriad of advances in technology for both treatment and detection, tumor dormancy and escape remains poorly understood. The biggest challenge is detecting and CTCs, solitary DTCs, and micro-metastases within hosts for studies, which can be as low as 1 per 10^5^ cells [106]. Current detection, for CTCs and DTCs is based on enrichment through size restriction or surface antigen recognition, both of which have limitations that may skew the actual concentration of CTCs and DTCs (reviewed in [107]), making it difficult to perform an accurate analysis or manipulate cells for detailed studies. Future studies directed on improving detection techniques and factors that prevent escape from tumor dormancy, in addition to direct targeting of dormant tumor cells, offer unique opportunities to achieve significant therapeutic gains.
